# Quarantine and Isolation during COVID‐19 outbreak: A case of online diagnosis of supraventricular arrhythmia through telemedicine

**DOI:** 10.1002/joa3.12431

**Published:** 2020-09-10

**Authors:** Anna Vittoria Mattioli, Matteo Ballerini Puviani, Andrea Malagoli

**Affiliations:** ^1^ Surgical, Medical and Dental Department of Morphological Sciences related to Transplant, Oncology and Regenerative Medicine University of Modena and Reggio Emilia Modena Italy; ^2^ Istituto Nazionale per le ricerche cardiovascolari U.O Modena Modena Italy

**Keywords:** arrhythmia, COVID‐19, quarantine, stress, Valsalva

## Abstract

The present case report highlights the usefulness of telemedicine during quarantine and isolation. The patient developed a supraventricular arrhythmia, and the diagnosis and management of the arrhythmia was done online.

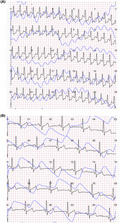

On March 2020 the WHO defined the diffusion of novel coronavirus, Severe Acute Respiratory Syndrome‐Coronavirus‐2 (SARS‐CoV‐2) as pandemic. As a consequence, the Italian Government among others has enforced quarantine on the population to contain the diffusion of the infection. Quarantine refers to the separation of communities who have been exposed to an infectious disease.

It is well known that quarantine is an unpleasant experience and is associated with several emotional disturbance: depression, stress, low mood, irritability, insomnia, and in the long term, post‐traumatic stress. Moreover people are worried about the possibility of contracting the infection and the closure of schools and business, increased this negative feeling.

From March 26 to April 26, several family groups of elderly individuals quarantined were selected and they voluntarily agreed to participate in the evaluation of self‐assessment of vital parameters.

The five WHO vital signs were measured twice a day using a noninvasive device called “ButterfLife^TM^” (VST srl, Modena Italy). ButterfLife records the five vital signs (temperature, oxygen saturation, respiratory rate, heart rate, and blood pressure) simultaneously and provide an output of behavior of the signs that can be easily analyzed by remote clinical staff using telemedicine support. ButterfLife is a system based on two main components: hardware for the acquisition of basic physiological signals (electrocardiogram, photopletysmography, temperature) and a signal processing algorithm, based on mathematical models, to derive the vital parameters (temperature, oxygen saturation, respiratory rate, heart rate, and blood pressure). The models have been validated through tests that will allow them to be certified and classified as a medical device. Every 5 seconds the software estimates the five vital parameters in addition to the signals visible on the ECG monitor and photopletysmography.

During this period a woman aged 77 suffering from previous episodes of paroxysmal atrial fibrillation reported palpitations and called nurse.

The ECG was sent to remote control center and the doctor diagnosed a supraventricular tachycardia (Figure 1A). The patient was instructed, by phone, to perform Valsalva maneuver at home and the ECG recorded few minutes after the maneuver showed the recovery of sinus rhythm (Figure 1B).

**Figure 1:**
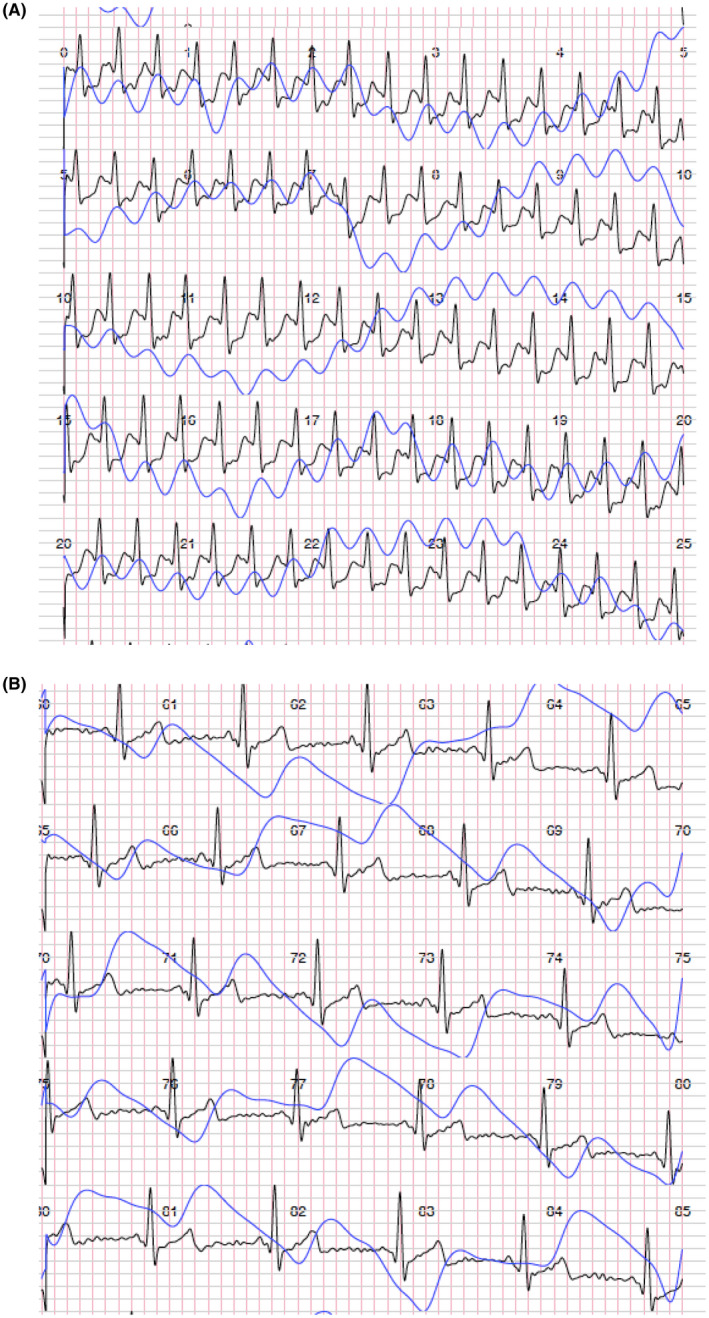
Panel A, Recording of supraventricular arrhythmia obtained by ButterfLife recording. It is a single trace record. The blue line represents photopletysmography record. Panel B, Single‐track ECG record after arrhythmia conversion. Sinus rhythm is evident

The COVID‐19 epidemic has resulted in a strong application of telemedicine tools. Due to the risk of infection, people are limited at home and in the case of the elderly, telemedicine was considered the best tool for home health monitoring. ButterfLife is a noninvasive, friendly device for self‐assessment of vital signs. This case report underlines the usefulness of a self‐monitoring friendly device in home evaluation. ButterfLife was tested used during a very stressful period of quarantine in two elderly couples living by themselves. The woman developed the arrhythmia immediately after receiving a phone call from her grandchildren whom she had not seen since the start of the quarantine. The call had deeply made her sad and fear about their health. It is well known that quarantine and isolation induce anxiety and stress. The relationship between stress and arrhythmias has been evaluated in several papers. Stress and anxiety have been found to act as a trigger of ventricular arrhythmias, which are found to be increased significantly after an earthquake.

Earthquakes may induce cardiac stress via sympathetic nervous system and the renin‐angiotensin‐aldosterone system axis activation, which can potentially lead to myocardial damage and other adverse cardiac effects. The acute stress response of an integrated cascade of physiological reactions has been well described.

The sympathetic nervous system has direct cardiac effects (positive chronotropy and inotropy effects via β1‐adrenergic receptors) and pressor effects (via α1‑adrenergic receptors), and in addition, affects the immune system. These mechanisms are known and widely explained in previous studies. We hypothesized that in this case the quarantine related stress plus the emotional stress of call acted together as a trigger for arrhythmia. The patient reported that she was very excited about her nephew's call and felt a sense of tachycardia; however, we do not have a record of the exact moment. The recording was activated a few minutes later following the appearance of symptom of tachycardia. Quarantine is an extreme situation to which people, especially the elderly, are not accustomed and together with the fear of contracting the disease, it has determined a chronic stress on which an acute traumatic trigger has grafted.

Little is known about the chronic stress induced by quarantine and isolation as it happens during COVID‐19 outbreaks. From 31 January to 2 February 2020, a study analyzed the initial psychological responses of the general public to the country’s outbreak of COVID‐19, and found that the 53.8% of respondents rating the psychological impact of the outbreak as moderate or severe. Individuals in isolation adopt an unhealthy lifestyle due to the difficulty of obtaining healthy food, the development of emotional eating, and the lack of physical activity. Unhealthy lifestyle promotes the development of arrhythmias both through metabolic alterations and through electrolyte dysfunction.

In conclusion, telemedicine and self‐monitoring increases consciousness and make subjects feel they are “doing something” for their own health, reducing stress associated with quarantine and isolation. In addition, monitoring vital signs allows an early diagnosis of arrhythmias and an early management of the disease.

## CONFLICT OF INTEREST

None Declared.

